# Impact of hypertension on cerebral small vessel disease: A post-mortem study of microvascular pathology from normal-appearing white matter into white matter hyperintensities

**DOI:** 10.1177/0271678X251333256

**Published:** 2025-04-12

**Authors:** Gemma Solé-Guardia, Anne Janssen, Rowan Wolters, Tren Dohmen, Benno Küsters, Jurgen AHR Claassen, Frank-Erik de Leeuw, Maximilian Wiesmann, Jose Gutierrez, Amanda J Kiliaan

**Affiliations:** 1 Department of Medical Imaging, Anatomy, Research Institute for Medical Innovation, Radboud University Medical Center, Donders Institute for Brain, Cognition & Behaviour, Preclinical Imaging Center PRIME, Radboud Alzheimer Center, Nijmegen, the Netherlands; 2 Department of Pathology, Research Institute for Medical Innovation, Radboud University Medical Center, Nijmegen, the Netherlands; 3 Department of Geriatrics, Research Institute for Medical Innovation, Radboud University Medical Center, Donders Institute for Brain, Cognition & Behaviour, Radboud Alzheimer Center, Nijmegen, the Netherlands; 4 Department of Cardiovascular Sciences, University of Leicester, Leicester, UK; 5 Department of Neurology, Research Institute for Medical Innovation, Radboud University Medical Center, Donders Institute for Brain, Cognition & Behaviour, Nijmegen, the Netherlands; 6 Department of Neurology, Vagelos College of Physicians and Surgeons, Columbia University Medical Center, New York, NY, USA

**Keywords:** Blood-brain barrier, hypertension, immunohistochemistry, inflammation, MRI, neuropathology, small vessel disease

## Abstract

Cerebral small vessel disease (SVD) is diagnosed through imaging hallmarks like white matter hyperintensities (WMH). Novel hypotheses imply that endothelial dysfunction, blood-brain barrier (BBB) disruption and neurovascular inflammation contribute to conversion of normal-appearing white matter (NAWM) into WMH in hypertensive individuals. Aiming to unravel the association between chronic hypertension and the earliest WMH pathogenesis, we characterized microvascular pathology in periventricular NAWM into WMH in post-mortem brains of individuals with and without hypertension. Our second aim was to delineate the NAWM-WMH transition from NAWM towards the center of WMH using deep learning, refining WMH segmentation capturing increases in FLAIR signal. Finally, we aimed to demonstrate whether these processes may synergistically contribute to WMH pathogenesis by performing voxel-wise correlations between MRI and microvascular pathology. Larger endothelium disruption, BBB damage and neurovascular inflammation were observed in individuals with hypertension. We did not observe gradual BBB damage nor neurovascular inflammation along the NAWM-WMH transition. We found a strong correlation between BBB damage and neurovascular inflammation in all individuals in both periventricular NAWM and WMH. These novel findings suggest that endothelium disruption, BBB damage and neurovascular inflammation are major contributors to SVD progression, but being already present in NAWM in hypertension.

## Introduction

Sporadic cerebral small vessel disease (SVD) affects the microvasculature in the brain parenchyma and is the major vascular contributor to dementia worldwide.^1–3^ Given the difficulty of visualizing the human microvasculature *in vivo*, the identification of SVD relies on magnetic resonance imaging (MRI) hallmarks,^4^ including white matter hyperintensities (WMH). The prevalence and burden of WMH increases with hypertension, being the most important risk factor for SVD.^5,6^ The mechanisms driving the conversion of areas at risk, such as normal-appearing white matter (NAWM), into WMH, remain largely unknown.

The pathogenesis of WMH is partly attributed to ischemia caused by hypertension-mediated arteriolosclerosis and endothelial dysfunction.^7,8^ Signs of arteriolosclerosis and endothelial dysfunction, such as Glucose transporter 1 (GLUT1) abnormalities,^9^ have not been characterized yet in the NAWM – WMH transition zone, the area in which progression of WMH occurs.^10^ More recently, evidence obtained with dynamic contrast-enhanced (DCE) MRI suggests that blood-brain barrier (BBB) leakage is among the earliest pathophysiological mechanisms in NAWM, and that it correlates to WMH burden.^11^ While this suggest a potential role for BBB disruption in the progression of WMH, no longitudinal study has shown yet that NAWM regions with increased BBB leakage have a greater likelihood of converting into WMH. The relation between BBB leakage and WMH progression needs further clarification.

Neurovascular inflammation is increasingly reported to be a major contributor to the progression of WMH in individuals with hypertension.^12^ Matrix metalloproteinases (MMPs), including MMP9 expressed by macrophages, have been identified as markers of neurovascular inflammation.^13,14^ Preclinical evidence also implies a role for MMP9 in BBB disruption,^15^ potentially through loss of tight junctions and neurovascular inflammation.^15–17^ However, pathological studies on the relation between markers of BBB damage (e.g., Immunoglobulin G extravasation^18^) and neurovascular inflammation in NAWM – WMH transition zone are lacking.

Therefore, we aimed to investigate the association between hypertension and microvascular pathology (microvascular endothelium and vessel wall damage, BBB damage, vascular remodeling and neurovascular inflammation) by studying periventricular NAWM and WMH in human post-mortem brains of individuals with hypertension and age-matched controls. Identifying these microvascular pathology processes in the context of hypertension may provide useful insights into the WMH progression. Secondly, we aimed to determine whether microvascular pathology may follow a continuum along the gradient of the periventricular NAWM into WMH. A promising approach to characterize the NAWM – WMH transition zone may be the integration of deep learning-based segmentation to define smaller segments from periventricular NAWM into the center of WMH with careful co-registration to immunohistochemical data.^19^ By characterizing the microvasculature within these segments we may potentially elucidate whether there is a gradual progression of microvascular pathology underlying these MRI abnormalities already in NAWM. Finally, we aimed to demonstrate the interplay between these emerging processes in post-mortem periventricular NAWM and WMH. Through a comprehensive assessment of the direct association among microvascular markers, focusing on BBB damage and perivascular inflammation, we may demonstrate that these processes follow a synergistic model, rather than contribute to WMH progression as isolated processes. Insights into the interplay of these emerging processes may pave the road for novel interventional therapies that address multiple facets of SVD underlying pathology simultaneously.

## Material and methods

### Cases

17 individuals with hypertension (according to guidelines at that time^20^) and 5 age-matched individuals with no clinical record of hypertension (control) were included in this study through the body donors’ program at the Radboud university medical center, Nijmegen, The Netherlands. Table 1 shows the demographic and clinical characteristics of the study cohort.

**Table 1.** Demographic and clinical characteristics of the study cohort.

	Total(n = 22)	Control individuals(n = 5)	Individuals with hypertension(n = 17)	*p* value
Demographics				
Age, mean ± SD, years	80.6 ± 8.1	80.2 ± 8.6	80.7 ± 8.2	*p = 0.905*
Sex, female, n (%)	10 (45.5%)	3 (60.0%)	7 (41.2%)	*p = 0.457*
*Post-mortem* delay, mean ± SD, hours	22.4 ± 6.8	20.0 ± 2.9	23.1 ± 7.4	*p = 0.375*
Risk factors				
BMI, mean ± SD, kg/m^2^	23.1 ± 4.4	23.8 ± 3.8	22.9 ± 4.7	*p = 0.756* ^*a*^
Diabetes, n (%)	5 (22.7%)	1 (20.0%)	4 (23.5%)	*p = 0.869*
Hypercholesterolemia, n (%)	11 (50.0%)	1 (20.0%)	10 (58.8%)	*p = 0.127*
Smoking, n (%)	7 (31.8%)	2 (40.0%)	5 (29.4%)	*p = 0.655*
Alcohol use, n (%)	3 (13.6%)	0 (0%)	3 (17.6%)	*p = 0.312*
Imaging				
Fazekas score, moderate to severe WMH (Score ≥2), n (%)	10 (45.5%)	0 (0%)	10 (58.8%)	p = 0.020*
WMH volume^b^, mean ± SD, mL	1.40 ± 0.68	0.91 ± 0.26	1.54 ± 0.70	p = 0.020*

BMI: body mass index; SD: standard deviation; WMH: white matter hyperintensity. *p < 0.05.

^a^Data missing for *n = *6 for BMI.

^b^These measures correspond to the periventricular WMH volume from the left hemisphere.

### Ethics approval and consent to participate

Measurements were performed on human post-mortem brains acquired via the body donors’ program at the Department of Medical Imaging, Anatomy of the Radboud university medical center (Radboudumc), Nijmegen, The Netherlands. All body donors in this program signed a written informed consent during their lifetime permitting the use of their body and parts for science and teaching. Anonymized medical records were provided with the transfer of the samples.

The study was subject to the Dutch Body Donation Act and performed within the framework of the Dutch body donation program.

All protocols concerning data acquisition and tissue processing were approved by the Medical Ethics Review Committee (Commissie Mensgebonden Onderzoek (CMO) region Arnhem-Nijmegen, The Netherlands, file No. 2017-3941), and are legislated under Dutch national law (BWBR0005009).

### Post-mortem MRI – acquisition and deep learning-based segmentation

**Figure 1.**
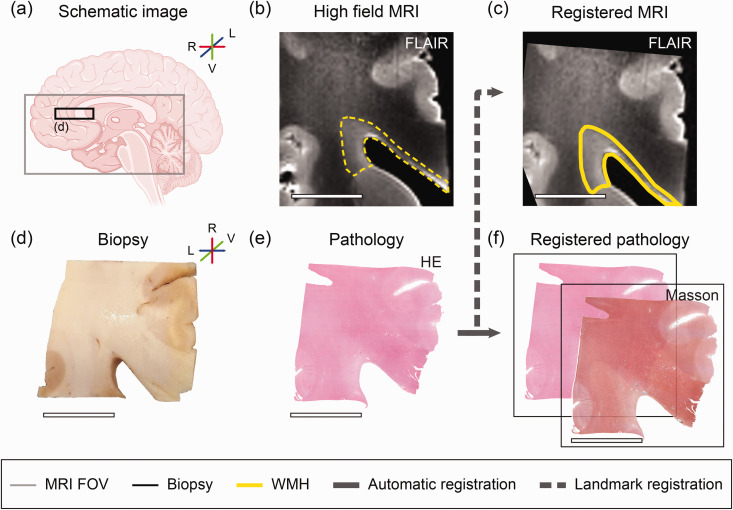
MRI and microvascular pathology study workflow overview. The left hemisphere was divided into ventral part for MRI scanning. (a) Schematic brain image. The grey square illustrates the dimensions of the ventral part of the left hemisphere. The black square illustrates the biopsy used for microvascular pathology stainings of approximately 2 × 2 × 0.5 cm. Sampling of the periventricular NAWM and WMH was successful for all individuals. (b) Image of corresponding MRI fluid-attenuated inversion recovery (FLAIR) axial slab of region of interest. White matter hyperintensity (WMH) before co-registration is outlined with a yellow dotted line. (c) Landmark-based MRI image registered to haematoxylin/eosin (HE). (d) Biopsy. (e) Histology section of haematoxylin/eosin (HE) and (f) Other immunohistochemistry sections were automatically registered to HE, including Masson’s staining. This figure was partly generated with Biorender.com. (L lateral, R Rostral, V Ventral) (scale bar = 1 cm).

Details on tissue processing (Figure 1) and post-mortem MRI data acquisition have been previously described.^12^ The left hemisphere was scanned post-mortem at room temperature on a Bruker 7 Tesla Clinscan MR system (Bruker Biospin, Ettlingen, Germany) interfaced with a Siemens Syngo VB15 console. The protocol included several sequences, including T1-weighted sequence (repetition time 20 ms, echo time 1 ms, voxel size 400 × 400 × 400 µm isotropic) and T2-weighted fluid-attenuated inversion recovery (FLAIR) sequence (repetition time 8200 ms, echo time 39 ms, 2 averages, voxel size 500 × 500 × 500 µm), which were used in this study.

In this study we segmented grey matter (GM), periventricular NAWM and WMH on FLAIR MRI, followed by further separation of these into smaller segments (1–3) based on FLAIR signal intensity to also visualize the NAWM – WMH transition zone from distal NAWM towards WMH center. Details on the data processing and model training have been previously described^19,21^ (code is available on GitHub).

### Immunohistochemistry

**Figure 2.**
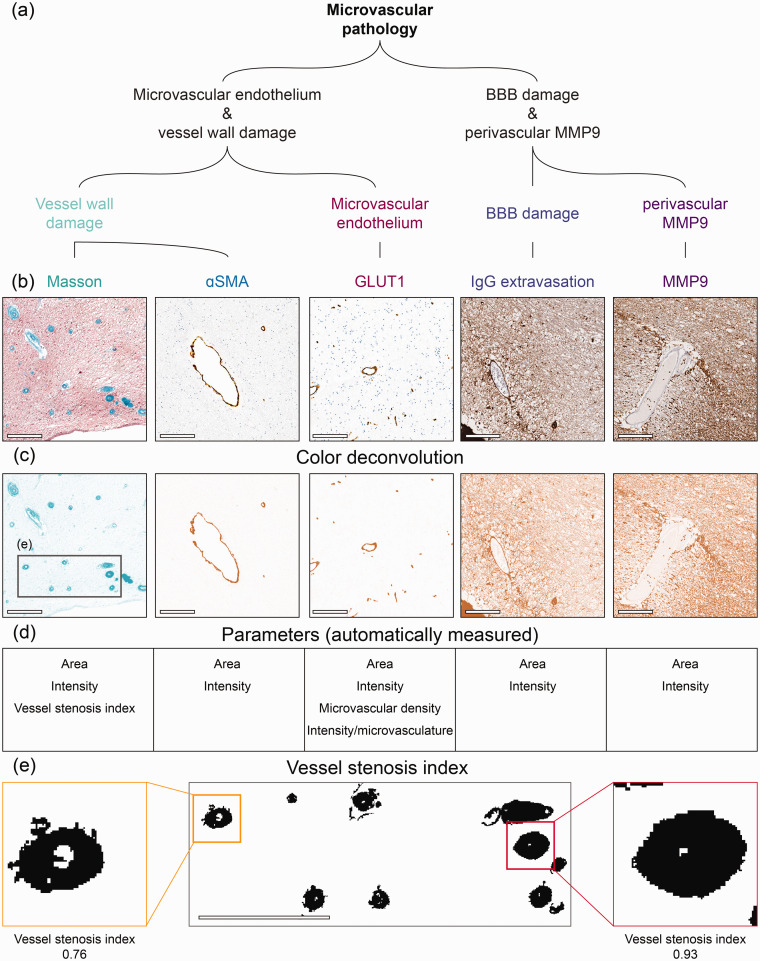
Immunohistopathological markers for microvascular pathology. (a) In this study we have included several stainings to examine microvascular pathology including microvascular endothelium, vessel wall and BBB damage, and neurovascular inflammation. (b) Shows close-up region from whole slide images for Masson, αSmooth Muscle Actin (αSMA), glucose transporter 1 (GLUT1), Immunoglobulin G (IgG) and matrix metalloproteinase 9 (MMP9) from left to right. (c) Outcome of color deconvolution for each of these stainings. Grey square represents the region shown in panel E. (d) List of parameters studied from each staining and (e) Representative region from Masson’s staining to illustrate the examination of vessel stenosis index. After color deconvolution and thresholding of the images, vessel stenosis index was automatically calculated by dividing the vessel wall area by their total area (vessel wall + lumen area) in ImageJ (scale bar = 200 µm).

Sections were stained following standard histological protocols for haematoxylin/eosin (HE) and Masson’s trichrome staining (Figure 2), referred as Masson hereafter. Masson was used to assess microvasculature collagen deposition within the vessel walls (collagenosis) of arterioles and venules. Immunohistochemistry was performed on adjacent sections for Glucose transporter 1 (GLUT1), αSmooth muscle actin (αSMA), Immunoglobulin G (IgG) and Matrix metalloproteinase 9 (MMP9) (Figure 2). GLUT1 is a glucose transporter protein expressed by endothelial cells that can be used to visualize blood vessels. GLUT1 has previously been demonstrated as a proxy for microvascular function since under ischemic conditions its expression is often increased to improve glucose uptake.^9,22–24^ It is known that endothelial cells are affected in SVD.^25^ Thus, GLUT1 may be used to quantify the presence of endothelial cells as an indicator of microvascular density. αSMA is a staining for vascular smooth muscle cells. IgG extravasation and MMP9 were used to examine degree of BBB damage and tissue remodeling/inflammation surrounding the microvasculature, respectively. The full immunohistochemistry protocol and antibody details are listed in the Supplemental material.

### MRI-pathology co-registration

Details on MRI-pathology co-registration have been described previously.^12^ Briefly, the MRI data (T1-weighted, FLAIR) were visually compared to the HE reference section to select the corresponding 2D MRI. MRI-immunohistochemical data was then co-registered based on this manual landmark selection (Figure 1(c) and (e)) using a custom written MATLAB script (MATLAB R2020a; MathWorks Inc., Natick, MA, USA).

### Detection and quantification of microvascular endothelium and vessel wall damage, BBB damage and perivascular MMP9

Stained GLUT1, αSMA and Masson sections were digitized using a Panoramic 1000 slide scanner (3DHISTECH Ltd, Hungary), while IgG and MMP9 sections were digitized using a Leica SCN400 Slide Scanner (Leica Biosystems, Wetzlar, Germany). All high-resolution digital images (0.25 µm/pixel) were exported and registered to corresponding HE sections as described elsewhere ^12^ (Figure 1(f)). After this multimodal registration, Masson, GLUT1, αSMA, IgG and MMP9 stained sections were imported and processed in ImageJ (version 1.54f, National Institute of Health, Bethesda, Maryland, United States) making use of the color deconvolution tool^26^ (Figure 2(b) and (c)). Immunohistochemistry sections were analyzed by using intensity threshold to isolate the target staining from the background. To account for varying staining intensities across individuals, the intensity threshold was determined for each staining by examining mean intensity values of positive stained control regions. Threshold settings based on the overall mean intensity were checked on individual basis.

To examine the underlying pathology of (increased) MRI signal intensity, we correlated voxel-wise FLAIR signal to immunohistochemistry for microvascular endothelium and vessel wall damage, BBB damage and vascular remodeling/neurovascular inflammation markers throughout deep learning-based periventricular WMH and NAWM segments for all individuals. In order to directly correlate MRI data with immunohistochemistry, all co-registered data images were divided into 0,16 mm^2^ (0.4 × 0.4 mm) pixels resembling voxel dimensions, and the average value per parameter within each pixel were calculated (Figure 2(d)). Relative stained area per pixel (0,16 mm^2^) hereafter referred as Area of the staining of interest and intensity – where higher values correspond to more pronounced staining amount – were calculated for all microvascular pathology stainings (Masson, GLUT1, αSMA, IgG and MMP9). Microvascular density was automatically counted by ImageJ based on the number of positive vessels after color deconvolution and thresholding (number per mm^2^) for GLUT1 stained sections. In addition, we calculated the ratio of intensity and microvascular density for GLUT1 stained sections. In Masson stained sections, vessel stenosis index was automatically calculated by dividing the vessel wall area by their total area (vessel wall + lumen area) in ImageJ (Figure 2(e)). Vessel stenosis index is similar to the manual sclerotic index.^27^ In our analysis, measures of BBB damage & perivascular MMP9 (IgG and MMP9) were included based on the presence of other microvascular markers within each pixel’s boundaries. Pixels with less than 50% of positive tissue were excluded from analysis. Finally, the average values within the included pixels was used to generate heatmaps.

### Statistics

Means and standard deviation (SD) were calculated for all continuous variables, as well as frequencies and percentages for categorical variables. Relationships between categorical variables were explored using the Chi-square test (χ^2^). We used multivariate analysis of variance (ANOVA) for group comparisons of continuous demographic variables and WMH volume. To address our first aim (investigate the association between hypertension and microvascular pathology), ANCOVA, controlled for age and fixation-immunohistochemistry interval, were used to compare microvascular pathology stainings – including microvascular endothelium (GLUT1), vessel wall (Masson, αSMA) and BBB damage (IgG), and neurovascular inflammation (MMP9) – between brains of individuals with hypertension and control individuals in periventricular NAWM and WMH. Results for GM can be found in *Supplemental material: Table S2.* In order to address our second aim (determine whether microvascular pathology may follow a continuum), ANCOVA, controlled for age and fixation-immunohistochemistry interval, were used to compare microvascular pathology stainings within periventricular NAWM and WMH segments in individuals with hypertension and control individuals. All ANOVA were performed with Bonferroni correction for multiple testing. Additionally, we tested for interactions between variables to ensure that our findings are robust and not driven by any specific region, and to determine if hypertension specifically impacts WMH or NAWM. When significant, p values of the variables were reported separately. In order to address our third aim (demonstrate whether there is an interplay between markers for microvascular pathology), we directly correlated and examined voxel-wise associations between MRI-based signal and microvascular pathology in periventricular NAWM and WMH segments using a Pearson correlation.

Statistical analysis was performed using IBM SPSS statistics 29 (IBM Corporation, Armonk, NY, USA). Alpha (2-tailed) was set at 0.05 for descriptive analysis and ANOVA, and at 0.01 for correlations.

## Results

Individuals’ characteristics are presented in Table 1. The brains of individuals with hypertension had a greater WMH burden than control individuals (p = 0.02; Table 1).

### The association between hypertension and microvascular pathology in periventricular NAWM and WMH

#### Microvascular endothelium and vessel wall damage

**Figure 3.**
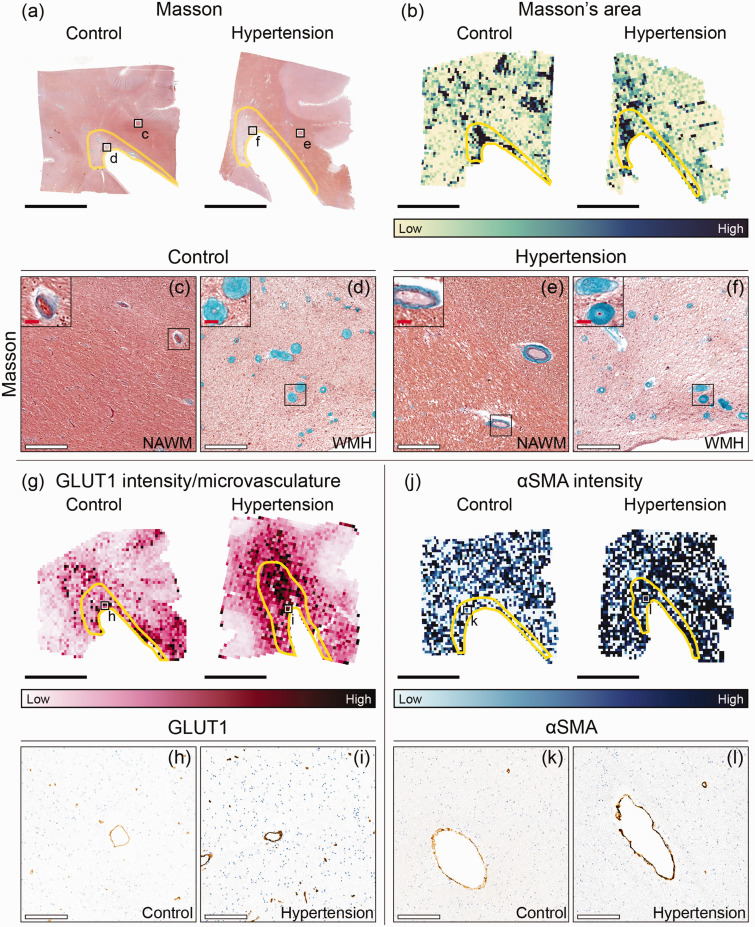
Immunohistopathological characterization of microvascular endothelium and vessel wall damage in individuals with hypertension and control individuals throughout periventricular white matter. Representative whole slide images of Masson’s staining of brains of normotensive individuals and individuals with hypertension (a) and their corresponding area heatmaps (b). The black squares (a) indicate regions of interest visible in C–F. The yellow outlines in image B depict white matter hyperintensities (WMH). Close ups (X20 magnification) are placed on the upper left corner of (c–f) and show the vessel wall thickening within WMH. G Representative glucose transporter 1 (GLUT1) intensity per microvasculature heatmaps of brains of normotensive individuals and individuals with hypertension. The black squares (g) indicate regions of interest visible in H,I. J Representative αSmooth Muscle Actin (αSMA) intensity heatmaps of brains of normotensive individuals and individuals with hypertension. The black squares (j) indicate regions of interest visible in k,l (black scale bar = 1 cm; white scale bar = 200 µm; red scale bar = 25 µm).

Vessel stenosis index assessed by Masson’s staining was 2.7% lower in periventricular NAWM and WMH of individuals with hypertension (0.638) compared to the control **group** (0.656; *p = 0.003;* Figure 3; Table 2). GLUT1 staining intensity (*p < 0.001;* Table 2) and GLUT1 intensity per microvasculature (*p = 0.004*; Figure 3), were larger in periventricular NAWM and WMH of individuals with hypertension compared to age-matched control individuals. Similarly, microvascular intensity of αSMA, a marker for vascular remodeling, was higher in periventricular NAWM and WMH of individuals with hypertension compared to age-matched control individuals (*p < 0.001;* Figure 3; Table 2*;* see *Table S2* for GM results).

**Table 2.** Microvascular pathology in individuals with hypertension and age-matched control individuals and periventricular WMH – NAWM.

		Control		Hypertension		p value^a^	
		NAWMmean ± SD	WMHmean ± SD	NAWMmean ± SD	WMHmean ± SD	Groups (hypertension vs. control)	ROIs (WMH vs. NAWM)
Microvascular endothelium and vessel wall damage							
Masson	Area (%)	0.35 ± 0.13	0.80 ± 0.39	0.31 ± 0.16	0.86 ± 0.59	p = 0.780	p < 0.001***
	Intensity (%)	31.7 ± 0.8	34.2 ± 2.0	31.8 ± 1.6	33.7 ± 2.2	p = 0.523	p < 0.001***
	Vessel stenosis index [0–1]	0.65 ± 0.04	0.66 ± 0.02	0.64 ± 0.02	0.64 ± 0.02	p = 0.003**	p = 0.725
GLUT1	Area (%)	0.77 ± 0.55	0.66 ± 0.49	0.91 ± 0.29	0.75 ± 0.29	p = 0.182	p = 0.145
	Intensity (%)	66.6 ± 7.2	66.6 ± 5.3	71.0 ± 3.7	69.8 ± 4.1	p < 0.001***	p = 0.591
	Microvascular density (microvasculature/mm^2^)	57.3 ± 20.6	50.1 ± 1.8	64.9 ± 16.8	47.8 ± 15.1	p = 0.189	p < 0.001***
	Intensity/microvasculature (a.u.)	5.8 ± 1.3	8.0 ± 1.2	7.1 ± 1.6	10.4 ± 2.7	p = 0.004**	p < 0.001***
αSMA	Area (%)	0.25 ± 0.04	0.23 ± 0.04	0.26 ± 0.08	0.28 ± 0.09	p = 0.336	p = 0.995
	Intensity (%)	58.9 ± 1.5	59.0 ± 1.5	61.0 ± 2.5	60.2 ± 2.7	p < 0.001***	p = 0.548
BBB damage & perivascular MMP9							
IgG	Area (%)	0.10 ± 0.08	0.53 ± 0.35	0.60 ± 1.23	0.99 ± 0.91	p = 0.043*	p = 0.063^#^
	Intensity (%)	59.9 ± 2.2	61.1 ± 3.1	59.5 ± 1.9	60.3 ± 2.5	p = 0.093	p = 0.018*
MMP9	Area (%)	2.72 ± 2.6	8.02 ± 4.06	2.09 ± 3.05	5.97 ± 5.11	p = 0.634	p < 0.001***
	Intensity (%)	38.0 ± 1.3	39.2 ± 2.0	40.4 ± 1.8	40.7 ± 2.3	p < 0.001***	p = 0.004**

αSMA: αSmooth muscle actin; GLUT1: glucose transporter 1; IgG: immunoglobulin G; MMP9: matrix metalloproteinase 9; NAWM: normal-appearing white matter; WMH: white matter hyperintensity. ^#^0.07<p ≤ 0.05, *p < 0.05, **p < 0.01, ***p < 0.001.

a p values represent values after Bonferroni correction.

For all individuals, area assessed with Masson’s staining was twofold higher in WMH (control: 0.80%; hypertension: 0.86%) than in NAWM **ROIs** (control: 0.35%; hypertension: 0.31%) (*p < 0.001;* Table 2). Masson’s staining intensity was larger in microvasculature located in WMH compared to NAWM (*p < 0.001*). Microvascular density assessed with GLUT1 was lower in WMH (control: 50.1 microvasculature per mm^2^; hypertension: 47.8 microvasculature per mm^2^) compared to NAWM (control: 57.3 microvasculature per mm^2^; hypertension: 64.9 microvasculature per mm^2^) (*p < 0.001;* Table 2). GLUT1 intensity per microvasculature was larger in WMH than in NAWM (*p < 0.001;* Table 2). These observations were consistent for all individuals, both hypertensives and controls.

#### Blood-brain barrier damage and perivascular MMP9

**Figure 4.**
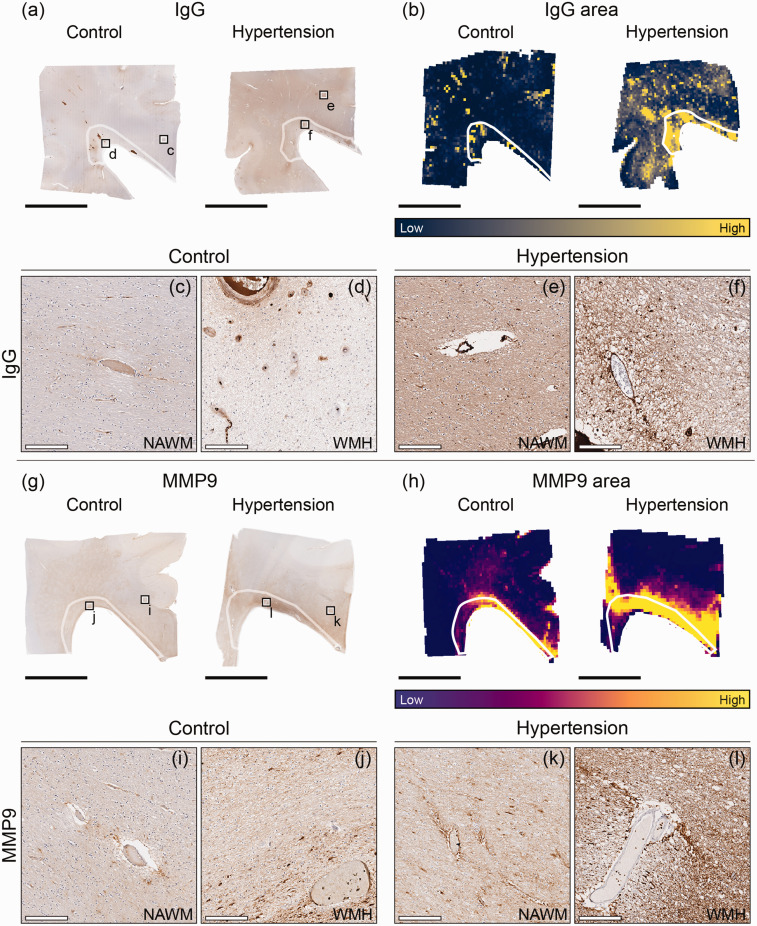
Immunohistopathological characterization of blood-brain barrier damage and perivascular MMP9 in individuals with hypertension and control individuals throughout periventricular white matter. Representative whole slide images of Immunoglobulin G (IgG) of brains of normotensive individuals and individuals with hypertension (a) and their corresponding area heatmaps (b). The black squares (a) indicate regions of interest visible in c–f. The white outlines in images B,H depict white matter hyperintensities (WMH). Representative whole slide images of matrix metalloproteinase 9 (MMP9) of brains of normotensive individuals and individuals with hypertension (g) and their corresponding area heatmaps (h). The black squares (g) indicate regions of interest visible in I–L (black scale bar = 1 cm; white scale bar = 200 µm).

Area of IgG staining extravasation as a marker for BBB damage was 2.5 fold in periventricular NAWM and WMH of individuals with hypertension compared to age-matched control **group** (*p = 0.043;* Figure 4; Table 2). The area of MMP9, as a marker for vascular remodeling and neurovascular inflammation, surrounding the blood vessels did not differ between individuals with hypertension and age-matched control individuals. Intensity of perivascular MMP9 was higher in periventricular NAWM and WMH of individuals with hypertension (*p < 0.001;* Figure 4; Table 2*;* see Table S2 for GM results).

For all individuals, perivascular IgG staining intensity was larger in WMH compared to NAWM **ROIs** (*p = 0.018;* Table 2). Area of perivascular MMP9 was 2.8 fold larger in the periventricular WMH compared to NAWM in individuals with hypertension and control individuals (*p < 0.001;* Table 2). Furthermore, intensity of perivascular MMP9 was 5% larger in WMH than in NAWM in individuals with hypertension and control individuals (*p = 0.004;* Table 2).

### Microvascular pathology along the periventricular NAWM into WMH

Only within WMH, and not in NAWM, we observed structural vessel walls abnormalities with Masson’s staining to the same extent in both hypertensives and controls along the **segments** (Table 3). Particularly, the vessels were significantly larger in the center of the periventricular WMH (1) – where voxel-wise FLAIR signal is the brightest – compared to the two adjacent WMH (2–3) segments (1 vs 2: *p = 0.039*; 1 vs 3: *p = 0.021;* Table 3). Similarly, Masson’s staining intensity was larger in the central periventricular WMH segment (1) compared to the (3) distal WMH border segment (1 vs 3: *p = 0.020;* Table 3). These observations were consistent for all individuals, both hypertensives and controls. For all individuals, we did not observe specific differences between periventricular WMH and NAWM segments for IgG nor perivascular MMP9 (Table 3).

**Table 3.** Microvascular pathology in individuals with hypertension and age-matched control individuals along the NAWM – WMH axis.

		NAWM			WMH			*p value* ^a^		
		Periventricular white matter segments			Periventricular white matter segments			Periventricular white matter segments		
		1mean ± SD	2mean ± SD	3mean ± SD	1mean ± SD	2mean ± SD	3mean ± SD	*(1 vs 2)*	*(2 vs 3)*	*(1 vs 3)*
Microvascular endothelium and vessel wall damage										
Masson	Area (%)	0.31 ± 0.14	0.33 ± 0.19	0.32 ± 0.21	0.61 ± 0.49	0.58 ± 0.38	1.27 ± 0.93	*p = 1.000* ^b^	p = 0.039* ^b^	p = 0.021* ^b^
	Intensity (%)	31.7 ± 1.5	32.0 ± 1.3	31.6 ± 1.5	33.3 ± 1.9	33.4 ± 2.1	34.9 ± 2.1	*p = 1.000* ^b^	*p = 0.141* ^b^	p = 0.020* ^b^
	Vessel stenosis index [0–1]	0.65 ± 0.02	0.64 ± 0.02	0.64 ± 0.03	0.64 ± 0.03	0.64 ± 0.03	0.63 ± 0.04	*p = 1.000*	*p = 1.000*	*p = 1.000*
GLUT1	Area (%)	0.85 ± 0.33	0.85 ± 0.34	0.93 ± 0.42	0.85 ± 0.29	0.73 ± 0.33	0.66 ± 0.38	*p = 1.000*	*p = 1.000*	*p = 1.000*
	Intensity (%)	69.8 ± 5.2	70.2 ± 5.4	69.9 ± 4.8	70.3 ± 4.1	69.6 ± 4.5	67.6 ± 4.7	*p = 1.000*	*p = 1.000*	*p = 1.000*
	Microvascular density (microvasculature/mm^2^)	62.0 ± 19.6	60.0 ± 18.5	67.5 ± 22.0	51.6 ± 16.5	46.9 ± 17.3	42.8 ± 18.7	*p = 1.000*	*p = 1.000*	*p = 1.000*
	Intensity/Microvasculature (a.u.)	6.8 ± 2.1	6.9 ± 1.7	6.6 ± 1.9	9.1 ± 2.3	10.3 ± 3.0	10.7 ± 3.8	*p = 0.612*	*p = 1.000*	*p = 0.956*
αSMA	Area (%)	0.25 ± 0.09	0.25 ± 0.06	0.27 ± 0.10	0.25 ± 0.10	0.24 ± 0.07	0.30 ± 0.16	*p = 1.000*	*p = 0.423*	*p = 1.000*
	Intensity (%)	60.7 ± 2.7	60.5 ± 2.2	60.3 ± 2.4	59.8 ± 2.8	60.0 ± 2.5	60.1 ± 2.5	*p = 1.000*	*p = 1.000*	*p = 1.000*
BBB damage & perivascular MMP9										
IgG	Area (%)	0.67 ± 2.02	0.40 ± 0.76	0.37 ± 0.58	0.71 ± 0.74	0.76 ± 0.77	1.19 ± 1.17	*p = 1.000*	*p = 1.000*	*p = 1.000*
	Intensity (%)	59.6 ± 2.1	59.6 ± 2.0	59.6 ± 2.0	60.1 ± 2.7	60.5 ± 2.7	60.9 ± 2.5	*p = 1.000*	*p = 1.000*	*p = 1.000*
MMP9	Area (%)	2.09 ± 2.77	2.23 ± 2.56	2.49 ± 3.77	6.79 ± 6.89	6.69 ± 4.99	5.82 ± 4.04	*p = 1.000*	*p = 1.000*	*p = 1.000*
	Intensity (%)	39.9 ± 2.2	39.8 ± 2.0	40.0 ± 1.8	39.9 ± 2.4	40.4 ± 2.6	40.8 ± 2.0	*p = 1.000*	*p = 1.000*	*p = 0.402*

NAWM and WMH [1–3] correspond to periventricular white matter segments, where higher segment numbers correspond to higher intensity FLAIR values. αSMA: αSmooth muscle actin; GLUT1: glucose transporter 1; IgG: immunoglobulin G; MMP9: matrix metalloproteinase 9; NAWM: normal-appearing white matter; WMH: white matter hyperintensity. ^#^0.07<p ≤ 0.05, *p < 0.05.

a *p* values represent values after Bonferroni correction.

b p values represent values across periventricular WMH segments. NAWM *p = 1.000.*

### MRI-microvascular pathology voxel-wise findings

#### MRI and microvascular pathology

Voxel-wise MRI signal throughout periventricular WMH and NAWM in individuals with hypertension and control individuals positively correlated with larger vessel walls (ρ = 0.405, *p < 0.001*) and Masson’s staining intensity (ρ = 0.403, *p < 0.001*), and GLUT1 intensity per microvasculature (ρ = 0.457, *p < 0.001*). Furthermore, voxel-wise MRI signal negatively correlated with microvasculature per mm^2^ assessed with GLUT1 staining in all individuals, both hypertensives and controls (ρ = –0.294, *p < 0.001*). For all individuals, voxel-wise MRI signal did not seem to correlate with IgG nor perivascular MMP9.

#### Microvascular pathology focusing on BBB damage and perivascular MMP9 correlations

In periventricular NAWM and WMH of individuals with hypertension and control individuals, we examined the correlation among voxel-wise IgG staining as a marker for BBB damage, and other microvascular pathology markers. We found that voxel-wise area of IgG positively correlated to αSMA intensity (ρ = 0.312, *p < 0.001*) and intensity of perivascular MMP9 in periventricular NAWM and WMH of individuals with hypertension and control individuals (ρ = 0.370, *p < 0.001*). We found that voxel-wise intensity of IgG staining in periventricular NAWM and WMH of individuals with hypertension and control individuals positively correlated to GLUT1 staining intensity (ρ = 0.238, *p < 0.001*) and area of perivascular MMP9 (ρ = 0.290, *p < 0.001*).

We also examined the degree of perivascular MMP9 as a marker for vascular remodeling and neurovascular inflammation, and other markers for microvascular pathology. We found that voxel-wise area of perivascular MMP9 in periventricular NAWM and WMH of individuals with hypertension and control individuals positively correlated with larger vessel walls (ρ = 0.323, *p < 0.001*) and Masson’s staining intensity (ρ = 0.242, *p < 0.001*). Voxel-wise intensity of perivascular MMP9 in periventricular NAWM and WMH of individuals with hypertension and control individuals positively correlated to αSMA staining intensity (ρ = 0.304, *p < 0.001*).

## Discussion

In this post-mortem study we characterized microvascular pathology in periventricular NAWM and WMH of individuals with hypertension and age-matched control individuals. As expected we observed that individuals with hypertension had a greater WMH burden compared to age-matched control individuals. While we found severe vessel stenosis index in all individuals, both hypertensives and controls, larger microvascular GLUT1 intensity per microvasculature in hypertensive individuals was observed; this may suggest compensatory reaction to ischemia.^22,23^ Notably, we observed larger perivascular MMP9 and IgG extravasation in periventricular NAWM and WMH of individuals with hypertension than in control individuals, indicating vascular remodeling/inflammation and BBB disruption. In both individuals with hypertension and in normotensive controls, vascular remodeling/inflammation and BBB disruption were larger in WMH. In addition, we observed lower microvascular density assessed with GLUT1 in WMH compared to NAWM in individuals with hypertension and control individuals suggesting abnormal vascular health in WMH across all individuals. For all individuals we observed gradual signs of vessel wall damage with Masson’s staining in periventricular WMH segments, but not in NAWM segments. Finally, voxel-wise determined IgG staining intensity correlated positively with perivascular MMP9 in periventricular NAWM and WMH in individuals with hypertension and control individuals, suggesting a close association between BBB damage and vascular remodeling/inflammation.

## The association between hypertension and microvascular pathology in periventricular NAWM and WMH

We found that all individuals showed severe signs of microvascular vessel wall damage beyond WMH as illustrated by a high degree of vessel stenosis index ^27^ with Masson’s staining. Contrary to our hypothesis, vessel stenosis index in periventricular NAWM and WMH was more elevated in control individuals compared to individuals with hypertension. Considering that the individuals in this study were of advanced age, and that aging can also contribute to vascular remodeling,^28^ independently or alongside hypertension, we cannot specify the reason behind this difference. Given that individuals with hypertension showed greater WMH burden despite the observation of a lower stenosis, our findings suggest that WMH pathogenesis is a complex multifactorial process beyond vascular remodeling.

Moreover, we observed a larger GLUT1 intensity per microvasculature in periventricular NAWM and WMH of individuals with hypertension. This novel finding builds upon prior in vitro and preclinical evidence of changes in GLUT1 expression under ischemic conditions.^22,23^ Therefore, the larger GLUT1 intensity per microvasculature in those with hypertension, may be a consequence of a compensatory mechanism aiming to maintain glucose supply upon reduced microvascular flow and/or glucose transport.^24^ Our findings showed that hypertensive individuals exhibit larger intensity of perivascular MMP9 in periventricular NAWM and WMH compared to age-matched controls. These findings, in line with previous research on vascular inflammation in SVD (reviewed by^29^) provide additional evidence linking neurovascular inflammation with SVD. While these processes were heightened in WMH compared to NAWM in all individuals, hypertension had comparable impact on both studied regions. Our findings demonstrate that microvascular pathology is significantly more pronounced in individuals with hypertension throughout the periventricular white matter.

Our study highlights several interesting findings regarding WMH underlying pathology in both hypertensives and controls. First, we observed lower microvascular density as indicated by GLUT1 staining in WMH compared to NAWM in all individuals. GLUT1 is a proxy for microvascular function.^9,22–24^ While previous studies have reported microvascular pathology in WMH,^3^ the occurrence of microvascular loss has been controversial. Our data shows that in SVD, microvascular density assessed with GLUT1 may be reduced in WMH, and therewith glucose transport.^30,31^ Although microvascular pathology exists in WMH regardless of blood pressure status, the larger severity in not only periventricular WMH, but also in NAWM among those with hypertension may be key to the pathogenesis of SVD.

## Microvascular pathology along the periventricular NAWM into WMH

Our previous research suggests that both periventricular NAWM and WMH segments based on MRI signal are able to capture a continuum of neurodegenerative processes regardless of blood pressure status, where the WMH center shows a greater degree of myelin loss.^21^ Here, area of vessel walls’ assessed with Masson’s staining was larger in the WMH center compared to adjacent segments within WMH in individuals with hypertension and control individuals. However, we did not observe gradual differences within periventricular NAWM segments regardless of blood pressure status. While this may seem in contrast to our previous observations on myelin pathology,^21^ the fact that gradual microvascular pathology was only captured within WMH indicates that NAWM and WMH segments may recapitulate later disease processes rather than fully reflect early microvascular endothelium damage or BBB damage. This suggests that microvascular pathology may occur at a finer spatial scale that cannot be resolved by these segments, highlighting the importance of exploring additional markers to detect early SVD pathogenesis in white matter.

## MRI-microvascular pathology voxel-wise findings

We showed that voxel-wise intensity of extravasated IgG positively correlated with perivascular MMP9 in periventricular NAWM and WMH of individuals with hypertension and control individuals, suggesting an overall interplay between BBB damage and neurovascular inflammation. MMPs are a family of endopeptidases involved in tissue remodeling and degradation of proteins in the extracellular matrix.^14^ MMPs expressed by macrophages, such as MMP9 are strongly associated with inflammation.^32^ In particular, MMP9 has been proposed to exacerbate inflammatory conditions.^33^ Amongst matrix metalloproteinases, MMP9 has been hypothesized to be involved in BBB breakdown.^34^ Since targeting neuroinflammation remains challenging due to its role in maintaining the central nervous system homeostasis,^35^ targeting neurovascular inflammation (e.g., inhibition of MMP9 downstream effects) could be a promising treatment strategy to reduce SVD progression. In individuals with hypertension, targeting neurovascular inflammation in combination with blood pressure management may prove particularly beneficial at stalling SVD progression. It is important to note that clinical trials on MMPs inhibition highlight short-term treatment as a more promising option, since medium and long-term trials have yielded mixed results (reviewed by^36^) Drugs like Miconazole,^37^ able to inhibit MMP9 downstream effects, e.g. MMP9-mediated vessel rupture, may prove beneficial in successfully reducing the progression of WMH, and ultimately dementia.

## Strengths and limitations

The strength of this study lies in its being the first comprehensive post-mortem assessment of microvascular pathology in individuals with hypertension and age-matched control individuals. Furthermore, we carefully co-registered MRI and immunohistochemical data, allowing an accurate characterization of the pathology from the periventricular NAWM into the center of periventricular WMH by analyzing smaller segments within NAWM and WMH, capturing the transition zone between NAWM and WMH. However, some limitations should be acknowledged. First, post-mortem studies are inherently cross-sectional, hindering our understanding of the temporal dynamics of processes involved in the pathogenesis of SVD. To further understand the dynamics between these processes, future research could benefit from longitudinal in vivo imaging of neuroinflammation and BBB leakage. Second, BBB damage was solely assessed post-mortem by examining the extent of IgG extravasation. Our findings support the association between BBB damage and WMH.^18,38^ However, in contrast with clinical observations,^39^ we did not find differences in IgG extravasation along the gradient from the periventricular NAWM towards the WMH visualized with smaller segments. This discrepancy may be attributed to the use of IgG as a marker for BBB damage because of its relative large molecular weight,^40^ which would require severe BBB damage to show IgG leakage into the parenchyma. However, there is no evidence that NAWM regions with BBB leakage convert into WMH. Future studies with other specific BBB markers such as tight junction proteins (e.g., Zonula occludens-1, Occludin)^30^ are needed to validate the extent of BBB disruption in greater detail.

## Conclusions

For the first time it is indicated due to our study, that microvascular pathology is also present in periventricular NAWM in individuals with hypertension, and not only in WMH. This study provides further evidence supporting the hypothesis that endothelium disruption, BBB damage and neurovascular inflammation is strongly associated with SVD pathogenesis in white matter. Future studies should investigate whether interventions targeting neurovascular inflammation, on top of antihypertensive treatment in individuals with hypertension, may reduce SVD progression.

## Supplemental Material

sj-pdf-1-jcb-10.1177_0271678X251333256 - Supplemental material for Impact of hypertension on cerebral small vessel disease: A post-mortem study of microvascular pathology from normal-appearing white matter into white matter hyperintensities Supplemental material, sj-pdf-1-jcb-10.1177_0271678X251333256 for Impact of hypertension on cerebral small vessel disease: A post-mortem study of microvascular pathology from normal-appearing white matter into white matter hyperintensities by Gemma Solé-Guardia, Anne Janssen, Rowan Wolters, Tren Dohmen, Benno Küsters, Jurgen AHR Claassen, Frank-Erik de Leeuw, Maximilian Wiesmann, Jose Gutierrez and Amanda J Kiliaan in Journal of Cerebral Blood Flow & Metabolism

## Data Availability

The datasets generated and/or analyzed during the current study are available upon reasonable request to the corresponding author.
