# Supplementary material for: Impact of hypertension on cerebral small vessel disease: A post-mortem study of microvascular pathology from normal-appearing white matter into white matter hyperintensities
Source: J Cereb Blood Flow Metab. 2025 Apr 12;45(9):1717–30. doi: 10.1177/0271678X251333256 (PMC11994636; doi:10.1177/0271678X251333256)
Supplement: sj-pdf-1-jcb-10.1177_0271678X251333256 - Supplemental material for Impact of hypertension on cerebral small vessel disease: A post-mortem study of microvascular pathology from normal-appearing white matter into white matter hyperintensities [file sj-pdf-1-jcb-10.1177_0271678X251333256.pdf]

Immunohistochemistry

Table S1 Information of antibodies used for immunohistochemistry in study

Antibody	Manufacturer	Catalogue #	Host	RRID	Dilution
**GLUT1**	Thermo Fisher Scientific, Waltham, MA, USA	RB-9052	Rabbit	AB_177893	1:400
**αSMA**	Sigma-Aldrich Corporation, Saint Louis, MO, USA	A2547	Mouse	AB_476701	1:7500
**IgG**	Abcam, Cambridge, UK	AB7159	Rabbit	AB_954878	1:300
**MMP9**	Sigma-Aldrich Corporation, Saint Louis, MO, USA	MABT531	Mouse	AB_94804	1:2000

αSMA αSmooth muscle actin, GLUT1 glucose transporter 1, IgG immunoglobulin G, MMP9 matrix metalloproteinase 9, RRID research resource identifier.

Sections were first deparaffinized in xylene, rinsed through graded ethanol series and demi water. For glucose transporter 1 (GLUT1) (complete antibody details in Supplementary Table 1), this step was followed by a 10 minute antigen retrieval step in Envision FLEX Target Retrieval Solution (K800421; Agilent, Santa Clara, CA, USA) at 97°C. Then, for GLUT1 and αSmooth Muscle Actin (αSMA), sections were processed further using a fully automated immunostainer (Lab Vision Autostainer 360; Thermo Fisher Scientific) and the EnVision FLEX visualization system (K8000, Agilent, Santa Clara, CA, USA, RRID: AB_2890017), according to manufacturer's instructions. Sections were rinsed in EnVision FLEX Wash Buffer (K800721-2; Agilent) for 5 minutes, followed by 5 minutes in Peroxidase-Blocking Reagent, and a 5 minute rinse in Wash Buffer. Sections were incubated with the primary antibody for 60 minutes. After incubation, sections were rinsed for 10 minutes in Wash Buffer and incubated for 15 minutes with EnVision FLEX+ Rabbit (LINKER) (K800921; Agilent) or Envision FLEX+ mouse (LINKER) (K802121; Agilent). After another 10 minute rinse in EnVision FLEX Wash Buffer, sections were incubated with EnVision FLEX HRP Solution (Agilent) for 30 minutes, then another 10 minute rinse in EnVision FLEX Wash Buffer. Sections were incubated with a mixture of EnVision FLEX 3,3'-diaminobenzidine (DAB) + and Substrate Solution (Agilent) for 10 minutes and rinsed in tap water for 10 minutes. All sections were counterstained using haematoxylin before dehydration using ethanol and xylene, and coverslipping.

Immunoglobulin G (IgG) and matrix metalloproteinase 9 (MMP9) sections were stained and processed as follows: For antigen retrieval, 1L of a 0.01M citrate buffer (pH = 6) was used. The slides were placed in a pressure cooker for 20 minutes at full power and remained in the pressure cooker at room temperature for another 15 minutes. Once the temperature was back to room temperature, the slides were washed with distilled water (dH2O) and in phosphate-buffered saline with Tween-20 (PBS-T) three times for 5 minutes each. Next, the slides were placed in a 3% hydrogen peroxide solution. Sections were permeabilized by incubation in 0.1% Triton-x-100 solution in PBS for 10 minutes, followed by 25 minutes 10% horse blocking serum. Sections were incubated with the primary antibody for 90 minutes. Sections were rinsed and a biotinylated horse anti mouse secondary antibody was added for 30 min, after which an ABC reagent was added for another 30 minutes. The 3,3'-diaminobenzidine (DAB) solution was added. As soon as the sections were developed, they were rinsed in dH2O. All sections were counterstained using haematoxylin before dehydration using ethanol and xylene, and coverslipping.

The association between hypertension and microvascular pathology in GM and NAWM

Microvascular endothelium and vessel wall damage

Vessel wall damage assessed by Masson's staining showed no differences between **groups**. GLUT1 staining intensity (*p = 0.049*) was larger in individuals with hypertension compared to age-matched controls in both GM and periventricular NAWM. In individuals with hypertension, αSMA staining was higher compared to age matched controls in both GM and periventricular NAWM (*p = 0.021*).

Table S2 Microvascular pathology in individuals with hypertension and age-matched controls and GM – periventricular NAWM

		Control		Hypertension		p value [a]	
		NAWM mean ± SD	GM mean ± SD	NAWM mean ± SD	GM mean ± SD	Groups (hypertension vs. control)	ROIs (GM vs. NAWM)
**Microvascular endothelium and vessel wall damage**							
Masson	Area (%)	0.35 ± 0.13	0.63 ± 0.29	0.31 ± 0.16	0.36 ± 0.26	p = 0.059 #	p = 0.039 *
	Intensity (%)	31.7 ± 0.8	30.0 ± 0.4	31.8 ± 1.6	30.6 ± 1.6	p = 0.524	p = 0.006 **
	Vessel stenosis index [0-1]	0.65 ± 0.04	0.64 ± 0.03	0.64 ± 0.02	0.62 ± 0.04	p = 0.189	p = 0.112
GLUT1	Area (%)	0.77 ± 0.55	1.45 ± 0.71	0.91 ± 0.29	1.24 ± 0.59	p = 0.883	p = 0.006 **
	Intensity (%)	66.6 ± 7.2	67.7 ± 5.2	71.0 ± 3.7	69.2 ± 3.7	p = 0.049 *	p = 0.836
	Microvascular density (microvasculature/mm^2)	57.3 ± 20.6	122.2±27.6	64.9 ± 16.8	111.0±51.6	p = 0.888	p < 0.001 ***
	Intensity/ microvasculature (a.u.)	5.8 ± 1.3	3.2 ± 1.1	7.1 ± 1.6	4.2 ± 2.6	p = 0.130	p < 0.001 ***

αSMA	Area (%)	0.25 ± 0.04	0.44 ± 0.10	0.26 ± 0.08	0.34 ± 0.11	p = 0.200	p < 0.001 ***
	Intensity (%)	58.9 ± 1.5	60.0 ± 0.4	61.0 ± 2.5	61.7 ± 2.4	p = 0.021 *	p = 0.240
**BBB damage & perivascular MMP9**							
IgG	Area (%)	0.10 ± 0.08	0.21 ± 0.13	0.60 ± 1.23	0.62 ± 1.74	p = 0.353	p = 0.892
	Intensity (%)	59.9 ± 2.2	60.2 ± 2.5	59.5 ± 1.9	59.9 ± 2.0	p = 0.629	p = 0.649
MMP9	Area (%)	2.72 ± 2.6	0.77 ± 0.79	2.09 ± 3.05	0.30 ± 0.45	p = 0.969	p = 0.109
	Intensity (%)	38.0 ± 1.3	38.6 ± 1.3	40.4 ± 1.8	41.6 ± 2.8	p = 0.007 **	p = 0.674

αSMA αSmooth muscle actin, GLUT1 Glucose transporter 1, GM grey matter, IgG Immunoglobulin G, MMP9 Matrix metalloproteinase 9, NAWM normal-appearing white matter. #0.07<p≤0.05, *p<0.05, **p<0.01, ***p<0.001

[a] p values represent values after Bonferroni correction

In GM of individuals with hypertension and age-matched controls, we found that Masson's staining intensity ($p = 0.006$) and area ($p = 0.039$) were lower compared to periventricular NAWM **ROI**. Furthermore, microvascular density assessed with GLUT1 was higher (113.5 ± 46.9 microvasculature per mm^2) compared to NAWM in all individuals (63.2 ± 20.0 microvasculature per mm^2) ($p < 0.001$). Similarly, GLUT1 area was larger in GM than periventricular NAWM in all individuals ($p = 0.006$). In all individuals, both hypertensives and controls, αSMA area was larger in GM compared to periventricular NAWM ($p < 0.001$).

Blood-brain barrier damage and perivascular MMP9

We did not observe groups differences in IgG staining extravasation as a marker for BBB damage in both GM and periventricular NAWM. In line with our findings in periventricular WMH, intensity of perivascular MMP9 was larger in those individuals with hypertension compared to age-matched control **group** in both GM and periventricular NAWM ($p = 0.007$).

For all individuals, we did not observe GM and periventricular NAWM (**ROI**) differences in IgG nor MMP9 stainings.
